# The competence-supportive and competence-thwarting role of athlete leaders: An experimental test in a soccer context

**DOI:** 10.1371/journal.pone.0200480

**Published:** 2018-07-11

**Authors:** Katrien Fransen, Maarten Vansteenkiste, Gert Vande Broek, Filip Boen

**Affiliations:** 1 Department of Movement Sciences, KU Leuven, Tervuursevest, Leuven, Belgium; 2 Department of Developmental, Personality and Social Psychology, University of Gent, Henri Dunantlaan, Gent, Belgium; Leibniz Institute for Educational Trajectories, GERMANY

## Abstract

The aim of this experiment was to study the growth-promoting and adverse impact of athlete leaders’ competence–supportive and–thwarting behavior on the motivation and performance of team members. Male soccer players (*N* = 144; *M*_*Age*_ = 14.2) were allocated to ad-hoc teams of five soccer players. These teams participated in two sessions, being randomly exposed to an athlete leader who acted either competence-supportive, competence-thwarting, or neutral during the second session. When the athlete leader was competence-supportive (versus competence-thwarting), his teammates’ intrinsic motivation and performance increased (versus decreased) compared with the control condition. The leader’s impact on intrinsic motivation was fully accounted for by team members’ competence satisfaction. These findings recommend coaches to invest in the competence-supportive power of their athlete leaders to establish an optimally motivating and performance-enhancing team environment.

## Introduction

Motivation is at the heart of a variety of activities, underpinning the choices that people make, their persistence in those choices, and the quality of the performed behavior [1]. The importance of motivation has been documented across a variety of contexts, including work, health care, and education [2]. In sport settings, the motivation of athletes has been linked to various beneficial outcomes such as concentration, sportspersonship, enjoyment, maintained effort, adherence, and performance (for a review, see [3]).

Although the critical role of sports coaches in fostering athletes’ motivation and performance has been extensively demonstrated, the role of athlete leaders has received far less attention. Athlete leaders are players within the team who are perceived as leaders by their teammates [4]. Besides the team captain (e.g., [5]), also informal leaders, who have no formal leadership status but acquire their leadership status through natural interactions with their teammates, can occupy important leadership roles (e.g., [6, 7]). In the current experimental study, we sought to examine whether athlete leaders can foster their teammates’ intrinsic motivation (i.e., engaging in an activity for its own sake, because it is interesting and enjoyable) and performance by supporting their sense of competence and effectiveness.

### Competence satisfaction as a facilitator of motivation and performance

According to the Self-Determination Theory [8, 9], competence is one of the three fundamental psychological needs that are essential to foster individuals’ intrinsic motivation and engagement. The need for competence refers to players’ natural keenness to gain mastery of tasks so that they feel increasingly skilled in what they do [10]. For instance, when athletes feel capable to meet the expectations of the coach or they have the feeling of making progress, they are said to have their need for competence met. Sports activities constitute a context in which feedback, either positive or negative, is provided continuously, thereby affecting athletes’ feelings of competence. Whether in trying to learn a new skill in soccer, in striving to become the best player in the school team, or in mastering a new technique in basketball, competence is apparent and influences individuals’ cognition, affect, motivation, and behavior [11].

Numerous studies have provided evidence for the critical role of competence satisfaction for fostering several favorable outcomes. For instance, in a sample of youth athletes from different team sport clubs, Jõesaar, Hein, et al. [12] found that athletes’ perceived competence satisfaction related positively to their intrinsic motivation, which, in turn, predicted their persistence. Furthermore, competence satisfaction of highly talented athletes has been found to relate positively to their well-being [13]. Moreover, in a longitudinal study with adolescent hockey players, Losier and Vallerand [14] reported that perceptions of competence predicted players’ intrinsic motivation over the duration of a hockey season.

Previous research [13] has demonstrated that a competence-supportive environment can be created (a) by offering challenging activities that match with athletes’ level of ability, (b) by expressing confidence in participants’ capacity to effectively engage in the activity; (c) by offering an effective model prior to task participation; (d) by providing encouragement and specific help during activity engagement; (e) by the presence of positive feedback and sincere praise after successful task completion; and (f) by the absence of critical and demeaning feedback after poor performance or mistakes. Such a competence-supportive environment was found to entail various beneficial outcomes, including motivation, team identification, cohesion, and performance [12, 15].

### Competence frustration as barrier to motivation and performance

To date, research almost solely focused on competence satisfaction and how to support the need for competence. However, not only can low competence satisfaction delay athletes’ growth, the presence of competence frustration (i.e., which is apparent when athletes’ need for competence is actively thwarted) can have severe adverse effects [16, 17]. In line with the distinction between need satisfaction and need frustration, Chen, Vansteenkiste, et al. [18] reported that the satisfaction of the need for competence uniquely contributed to individuals’ well-being, whereas the frustration of this need uniquely contributed to individuals’ ill-being.

Although addressed in theoretical overviews (e.g., [16]), need thwarting (i.e., the active undermining of athletes’ psychological needs) as well as the resulting need frustration (i.e., the subjective experience of failure and inadequacy in the case of competence frustration) remain understudied areas in the sports context, although the work by Bartholomew and colleagues forms a notable exception [17]. Nevertheless, sport constitutes an interesting research setting to study both competence satisfaction and frustration because athletes experience both support and encouragement as well as pressure and critique from multiple sources, including coaches [17], parents [19, 20], but also teammates. To examine whether supporting athletes’ competence promotes growth, whereas thwarting their competence undermines their functioning, an experimental study was set up involving a competence-supportive, neutral, and competence-thwarting condition. In contrast to previous studies, which mainly focused on competence support (e.g., [21, 22]), the present study is the first to experimentally examine the impact of both competence support and competence thwarting by the leader.

### The role of athlete leaders

In previous motivation research, primary attention has been devoted to the extent to which motivating style of authority figures (e.g., manager, teacher, or coach) influences their followers (i.e., employees, students, or athletes respectively) (e.g., [13, 23, 24]). By contrast, the motivating influence of peers, who stand in a less vertical and more horizontal relation to their teammates, has been largely neglected.

Nevertheless, a few studies inspired by SDT provide promising evidence for the motivating role of peers above and beyond the motivating role of authority figures. The first study demonstrated in a hospital setting that the motivating behavior of fellow patients was even more predictive for patients’ changes in motivation over the course of treatment than the motivating style of staff members [25]. In addition, Moreau and Mageau [26] observed in an organizational setting that the motivating behavior of colleagues was more predictive for employees’ work satisfaction and well-being than the motivating style of their supervisors.

Also in sport settings, primary research attention has been devoted to the influence of the coach, which holds a more vertical relation to the athletes. Recently, a growing interest has emerged in the impact of athlete leaders, namely those athletes who fulfill a leadership role in the team (for a review, see [27]). For instance, leadership quality within the team was found to affect team members’ identification with the team, their team confidence, and the team’s task and social cohesion [28–30].

Further, Fransen, Haslam, et al. [31] examined the role of competence support and competence thwarting by the athlete leader. During an experimental study, teams of four basketball players completed a shooting task twice. Each team was complemented by a research confederate, who acted as the athlete leader of the team and who either expressed high or low confidence in the athletes’ abilities to win the competition. The expression of high or low confidence constituted a way to optimize or undermine team members’ feelings of competence. When the athlete leader expressed high (rather than low) confidence in the team, the other team members were more confident in the team’s abilities and also their performance improved gradually during the test session. However, athletes’ intrinsic motivation was not included as an outcome and, more importantly, no control group was included.

### The present study

Given that the majority of studies in the field of sport research have focused on the role of sports coaches, the role of athlete leaders to motivate their team members has been underexplored. Because athlete leaders are part of the team and in close proximity of the other players on the field, they have the potential to strongly influence their teammates, both in a positive and in a negative way. The aim of the present study was therefore to experimentally study the potential growth-promoting and adverse impact of athlete leaders on the motivation and performance of fellow team members. Specifically, we examined whether athlete leaders affect teammates’ satisfaction of their need for competence and, as a result, influence their intrinsic motivation and performance.

Although some experimental work has been conducted that focuses on competence support in educational environments (e.g., [13]) or in laboratory settings (e.g., [22, 32]), experimental research in sport settings is rare (e.g., [21, 31]). Moreover, to examine whether any difference between a competence-supportive and a competence-thwarting athlete leader can be ascribed to the facilitative effect of competence support or to the debilitative effect of competence thwarting or both, a control condition is required. Such a control group was missing in the experiment of Fransen, Haslam, et al. [31].

Furthermore, previous studies have not taken into account the interactive nature of team sports. For example, previous research has mainly focused on the impact of athlete leaders on individual athletes (e.g., [29]), and also the performance task used by Fransen, Haslam, et al. [31] required little interaction between players. To obtain more insight in these team dynamics, we opted therefore for an experimental design in which ad-hoc soccer teams engaged in two tasks with variable degrees of interaction, that is, a dribbling-shooting task, which required only little interaction so that individual performance could be tracked, and a passing task, which involved a lot of interaction and, hence, only the team performance could be tracked. In line with previous research by Mouratidis, Vansteenkiste, et al. [33] in an educational environment, we expected that the positive effects of competence support and the negative effects of competence thwarting would be apparent both at the individual and at the team level.

More specifically, the following three hypotheses were examined. First, we hypothesized that competence support by the athlete leader would increase players’ competence satisfaction (H1a), players’ intrinsic motivation (H1b), and their performance (H1c) relative to both the control and competence-thwarting condition. By contrast, we expected that the thwarting of athletes’ competence would reduce athletes’ competence satisfaction (H2a) and would undermine their intrinsic motivation (H2b) and performance (H2c) relative to the control group. Second, we expected that both the growth-promoting role of competence support and the debilitating impact of competence thwarting on intrinsic motivation (H3a) and performance (H3b) could be accounted for by players’ increased or decreased competence satisfaction, respectively. Furthermore, we will extend previous work by not only focusing on the individual level, but testing the hypotheses at the team level as well.

## Method

### Procedure

The presidents of 11 Flemish soccer clubs and the organizers of two youth soccer camps were contacted to participate in the experiment in the period between February and May 2014. Five clubs and one organizer of a soccer camp agreed to participate, yielding a response rate of 46%. Three clubs did not respond to our invitation. The remaining three clubs and soccer camp did not fulfill one or two of the study-entry eligibility requirements: (1) a targeted age range between 12 and 17 years; and (2) co-occurring training sessions of different participating teams at the same location, so that the experimental groups could be composed of players of different teams.

After confirming their participation, a research assistant attended a training session of the club or soccer camp. After introducing himself, he obtained informed consent from all participants and full confidentiality was guaranteed. The research assistant divided the participants in experimental groups of four players. Each of these groups was complemented by a research confederate (who pretended to be one of the players). Together, the team completed the requested tasks out of sight of the remaining players. Each experimental session (including four participants and one confederate) lasted about 45 minutes. No players withdrew their participation during the experiment.

The ethics committee of KU Leuven, Belgium, approved this study. Furthermore, written informed consent was obtained from all participants before they started filling in the questionnaire. Furthermore, participants were guaranteed confidential treatment of their answers and they were informed that their participation was voluntary and that they could withdraw their participation at any time. Directly after the experiment, a debriefing took place during which participants were informed about the conducted manipulations and the aim of the experiment. In addition, after the full data collection was completed, participants were informed about the performance ranking of all participating teams, as well as about the scientific findings and implications of the study.

The data of the present experimental study have been used in one other manuscript [34], which used the Social Identity Approach to Leadership [35] as a theoretical framework to examine athlete leaders’ impact on the team functioning. More specifically, the study findings revealed that athlete leaders influenced teammates’ team confidence, with team identification and the leader’s perceived identity leadership behavior being the underpinning mechanisms. It should be noted however that the Social Identity Approach to Leadership provides only one angle to explain athlete leaders’ impact on the team functioning. Therefore, in the present article, we decided to use the Self-Determination Theory [9] as the guiding theoretical framework to test the impact of athlete leaders’ competence support/thwarting on teammates’ intrinsic motivation, with competence satisfaction being the underpinning mechanism. The measures of perceived competence support/thwarting, competence satisfaction, and intrinsic motivation were not used in the previous manuscript.

### Participants

In total, 144 male soccer players participated in our experiment. The soccer players were on average 14.2 years old (12–17 years old; *SD* = 1.1) and had 7.9 years of soccer experience (*SD* = 2.3). Participants were divided into 36 groups of four players. As mentioned before, each experimental group consisted of players from different teams to avoid that players would be familiar with each other, thereby increasing the experiment’s internal validity.

### Experimental design

#### Procedure

Players of the same experimental team received an identical soccer shirt to foster players’ identification with their newly-assembled team. Furthermore, each team was complemented by one of two male confederates, who were unknown to the players and who alternatively served as the athlete leader of the different teams. To ensure that the team members would perceive the confederate as their leader, he was introduced as the captain of the team. Furthermore, because previous studies [36–38] revealed that age and competence are typical characteristics of athlete leaders, the assigned confederate was on average six years older and played at a national level. To further consolidate the confederate’s leadership status, the team participated in a short soccer quiz before starting the actual test sessions. Because the confederate was already informed on the correct answers beforehand, he was able to lead the team discussions and to demonstrate his soccer knowledge, such that his leader status got further strengthened. In the remainder of the text the confederate will be termed ‘athlete leader’.

Each team subsequently completed two similar test sessions: the first session represented a baseline assessment and the second session represented the actual experimental manipulation. To guarantee that participants would exert their maximum effort in both sessions, they were informed that the scores of both test sessions would be aggregated to obtain an overall team score. Each of both test sessions included a passing and a dribbling-shooting task.

#### Passing task

**Fig 1:**
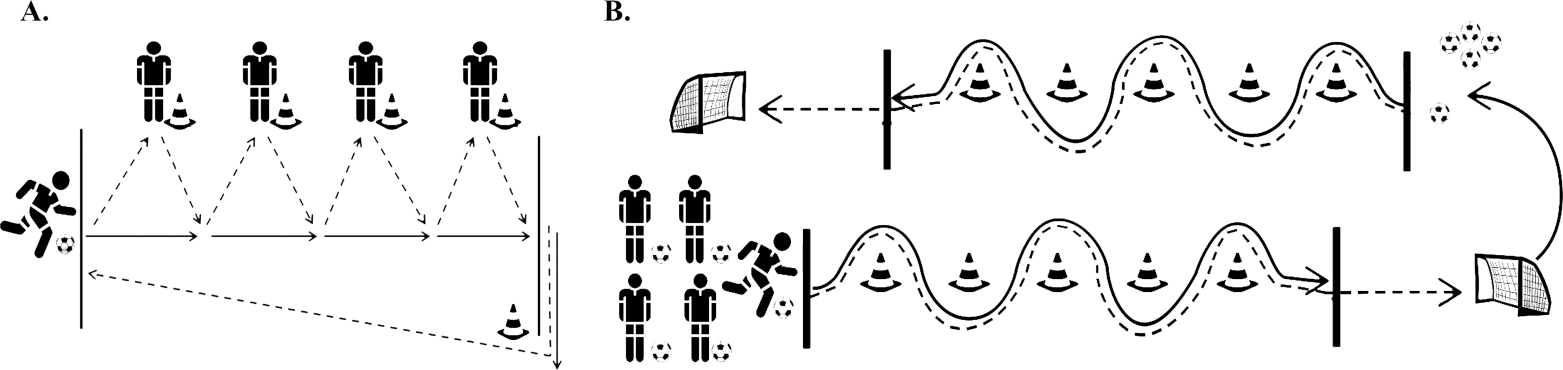
Schematic representation of (A) the passing task and (B) the dribbling–shooting task. Solid lines represent the movement pattern of the players, while the ball movement is represented by the dashed lines.

The passing task (Fig 1A) was highly interactive in nature. More specifically, the athlete leader started the exercise by passing the ball to the second player in line, who immediately passed the ball back. The same procedure was repeated with the three other players in the team, after which the athlete leader passed the ball back to the second player, who had meanwhile moved to the starting line to start the exercise anew. All players then moved one cone to the right, so that the athlete leader could occupy the last cone. The team successfully completed the task when each player in the team had completed the passing task four times, adding up to 20 rounds in total. The goal set for the team was to complete the task as fast as possible. To minimize learning effects, the team performed a trial before starting the first test session, so that every player understood the task well beforehand.

#### Dribbling-shooting task

The dribbling-shooting task (Fig 1B) required less interaction between the players so that both athletes’ individual and team performance could be taken into account. The athlete leader started the exercise by dribbling the ball between five cones, after which he tried to shoot a goal, demarcated by two cones. This goal attempt had to be taken from behind the marked line (see Fig 1B). Subsequently, the athlete leader took a new ball and completed the same exercise in the other direction. As soon as he touched the hand of the second player, this player could start the exercise. The exercise was completed when each player had performed the exercise four times, adding up to 20 rounds in total. Similar to the passing task, this task also started with a trial to ensure all players clearly understood the task.

### Manipulation

In the present experiment, we manipulated the *behavior of the athlete leader*, and more specifically the extent to which the athlete leader supported or thwarted his teammates’ competence. We adopted a 3 x 2 design, with the behavior of the athlete leader as between-subject variable (i.e., three experimental conditions) and time as within-subject variable (i.e., two different test sessions). The first test session involved a baseline assessment, in which the leader acted in a neutral manner. During the second test session, the athlete leader’s behavior was experimentally manipulated: the athlete leader supported teammates’ competence in 12 randomly selected teams, he acted neutrally in 12 other randomly selected teams (i.e., control condition), and he actively thwarted teammates’ competence in the remaining 12 teams.

With respect to our experimental manipulation, we should note that competence support is a fairly broad construct that encompasses different practices, such as the provision of positive informational and motivational feedback, the expression of team confidence, and the creation of a structured environment by providing clear guidelines and expectations [11, 39, 40]. In the present study, a well-structured environment was created by using a predefined soccer task and by providing clear guidelines and expectations. The specific facet of *competence support* that we manipulated in the present study is the extent to which athlete leaders provided positive feedback and encouragement to their teammates (e.g., “Great passing. Keep going!”, “Nice ball control!”, or “Great play, team!”) and the extent to which the leaders expressed confidence in their teammates’ abilities to effectively perform the tasks (e.g., “Great play, team. Keep it up and we will easily win this soccer contest!”). Finally, to match the verbal expression of the athlete leader, he also demonstrated positive body language, thereby displaying signs of enthusiasm and confidence.

To establish a *competence-thwarting environment*, the athlete leader behaved in exactly the opposite manner. That is, the leader provided critical feedback when his teammates performed poorly or made a mistake (e.g., “Your level of performance is really poor, even my grandma could do better”; “I don’t call this soccer anymore, this is hopeless.”). Furthermore, the leader indicated that he lost confidence in his team, both through his body language (e.g., groaning, hanging his head and shoulders) and by verbal expressions (e.g., “With this team, we can never win this contest. Do we really have to keep on playing?”). To standardize our manipulation, a detailed script was outlined for each of the experimental conditions, including the specific actions and their frequency that the leader was asked to perform, such that the number of competence-supportive and competence-thwarting statements was equal across conditions. In the control condition, the leader acted neutrally and provided no competence-related feedback.

### Measures

Participants completed the same two-page questionnaire after both the first and second session.

#### Perceived leader status

To examine whether the confederate was perceived as athlete leader of the team, participants answered the following question “To what extent do you perceive each of your teammates to be the leader of your team?” on a scale, ranging from -3 (*not at all*) to 3 (*completely*). We then compared the perceived leader status of the appointed leader to the status of the other players.

#### Manipulation check

To determine the effectiveness of the competence manipulation, we assessed participants’ perceived competence support of the leader using four items which were proposed by Standage, Duda, and Ntoumanis [41]. Each of the items was assessed on a 7-point Likert scale ranging from -3 (*strongly disagree*) to 3 (*strongly agree*). An example item is: “During this soccer test, our captain made us feel like we were able to successfully perform the requested tasks.” The Cronbach’s alpha of this 4-item scale was .88 and .94 after the first and second session, respectively, revealing an excellent internal consistency.

#### Competence satisfaction

To assess players’ competence satisfaction, we used the Need Satisfaction Scale (NSS) introduced by La Guardia, Ryan, Couchman, and Deci [42]. The NSS contains three items, each preceded by the stem ‘In this soccer team…’, and each item was scored on a 7-point Likert scale ranging from -3 (*strongly disagree*) to 3 (*strongly agree*). An example item of this scale is: “In this soccer team, I feel like a competent soccer player.” The internal consistency of the present 3-item scale was excellent, as demonstrated by a Cronbach’s alpha of .85 and .92 after the first and second session, respectively.

#### Intrinsic motivation

To assess participants’ intrinsic motivation, we used the 4-item intrinsic motivation subscale of the Sport Motivation Scale-6 [43]. Each of the items was scored on a 7-point Likert scale, anchored by -3 (*strongly disagree*) and 3 (*strongly agree*). An example item of this scale is: “I did my best during this soccer test because of the excitement I felt when I was really involved in what happened on the field.” The internal consistency of the present scale was .77 and .89 after the first and second session, respectively.

#### Performance

The rather individual nature of the dribbling-shooting task allowed us to extract an individual performance indicator by recording the time that each individual player needed to complete the exercise twice (back and forth). To obtain the total individual performance score for this task, we aggregated each player’s individual time across his four consecutive trials, with a faster exercise performance being indicative of a better performance. The total time the team needed to complete the task (i.e., 10 rounds back and forth) was used as a performance indicator at the team level. The highly interactive nature of the passing task only allowed extracting a performance indicator at the team level for this task by recording the total time that the team needed to complete the passing task.

In addition, we calculated a total performance indicator at the team level across both tasks by adding the team-level performance indicators of both dribbling-shooting task and passing task (i.e., the time the team needed to complete both exercises). In order to control for a possible effect of the athlete leader’s performance, we omitted the time of the athlete leader in the dribbling-shooting task. In the passing task, in which no individual performances could be isolated, we asked the athlete leader to perform the task as well as possible during both test sessions, regardless of the experimental condition.

## Results

The complete datasets, both at the individual level and at the team level, can be found in the attached datasets, S1 Table and S2 Table, respectively.

### Preliminary analyses

#### Perceived leader status

Compared with the average leader status of all other players averaged across all teams (*M* = 1.29; *SD* = 1.16), the leader status of both research confederates who acted alternately as an athlete leader was significantly and substantially higher (*M* = 2.57; *SD* = .72 for the first confederate and *M* = 2.15; *SD* = .95 for the second confederate). This result suggests that our confederates were clearly perceived by their teammates as the athlete leader in the team. Because a Shapiro-Wilk test revealed that the distribution of the leader status of both the athlete leader and the participants deviated significantly from the normal distribution (*p* < .001), the non-parametric Wilcoxon Signed Rank test was used. The effect size *r* was calculated by dividing the standardized test statistic *z* by the square root of the total number of observations. The results confirmed that the athlete leader was perceived to have significantly greater leader status than all remaining players (*r* = .51; *p* < .001), pointing at a large effect [44]. More detailed analyses revealed that, before the second test session, the athlete leader was perceived as the person with the highest leader status in 30 of the 36 teams. In the six remaining teams, the difference between the perceived leadership quality of our confederate and the perceived leadership quality of the best leader in the team did not exceed .25 scale points on a 7-point scale. This means that besides the confederate, also an informal leader was perceived as athlete leader in these six teams.

#### Manipulation check

Table 1 presents the means, standard deviations, and correlations between all the included variables. To compare the perceived competence support provided across the three experimental conditions, we conducted a 3 x 2 repeated measures ANOVA, with experimental condition (competence support vs. neutral vs. competence thwarting) as between-subjects factor and time as within-subjects repeated measure (second vs. first test session). The results, which are displayed in Table 2, revealed a significant interaction effect. Moreover, the post hoc analyses (i.e., 2 x 2 repeated measures ANOVAs) indicated significant interaction effects for each pair of experimental conditions. Specifically, the perceived competence support by the leader was found to significantly increase in the competence-supportive condition (*t* = 4.11; *d* = .61; *p* < .001), to stay stable in the control condition (*t* = .96; *d* = .15; *p* = .34), and to significantly decrease in the competence-thwarting condition (*t* = 5.85; *d* = .89; *p* < .001), with the effect size Cohen’s *d* (i.e., standardized difference between two means, expressed in standard deviation units) pointing at a large effect in the competence-supportive and -thwarting condition [44]. These findings confirm that the manipulation of the competence support by the athlete leader (support vs. neutral vs. thwarting) was successful.

**Table 1:** Means, standard deviations, and correlations between all the included variables.

	*M*	*SD*	1.	2.	3.	4.	5.	6.	7.	8.	9.
1. Perceived competence support at T1	1.60	1.02									
2. Perceived competence support at T2	1.27	1.72	.53***								
3. Competence satisfaction at T1	1.66	1.05	.67***	.41***							
4. Competence satisfaction at T2	1.76	1.25	.63***	.63***	.65***						
5. Intrinsic motivation at T1	1.88	.89	.66***	.29**	.60***	.51***					
6. Intrinsic motivation at T2	1.79	1.11	.68***	.56***	.59***	.69***	.62***				
7. Objective individual performance at T1	76.49	8.92	.01	.19*	.01	.08	-.01	.05			
8. Objective individual performance at T2	74.01	8.43	-.11	.18*	-.06	.04	-.14	-.05	.75***		
9. Objective team performance at T1	295.50	39.82	-.10	.15	-.13	-.08	-.13	-.10	.57***	.67***	
10. Objective team performance at T2	284.53	42.96	-.20*	-.02	-.21*	-.15	-.19*	-.18*	.53***	.67***	.95***

*p < .05

**p < .01

***p < .001.

*Note*. The performance measures were assessed as time measures (seconds). The individual performance measures (7 and 8) were assessed in the dribbling shooting task, whereas the team performance measures (9 and 10) were assessed in passing task.

**Table 2:** The findings of 3 x 2 repeated measures ANOVAs for all outcome variables with time (second vs. first test session) as the within-subjects repeated measure and the experimental condition (CS vs C vs CT) as the between-subjects factor, including the results of the post hoc analyses of the interaction effects.

	M at Time 1 (SD)	M at Time 2 (SD)	Main time effect F (η^2^) (*df* = 1)	Main condition effect F (η^2^) (*df* = 2)	Interaction effect time x condition F (η^2^) (*df* = 2)	*Post hoc analyses for interaction effect*		
						*F (CS-C)* (η^2^) (*df* = 1)	*F (CT -C)* (η^2^) (*df* = 1)	*F (CS-CT)* (η^2^) (*df* = 1)
**1. Perceived competence support of the leader**			12.08** (.09)	33.80*** (.35)	37.69*** (.37)	6.07* (.07)	32.36*** (.28)	49.82*** (.35)
A. Competence support (CS)	2.05 ± .73	2.49 ± .45						
B. Control (C)	1.41 ± 1.10	1.50 ± 1.25						
C. Competence thwarting (CT)	1.36 ± 1.03	-.24 ± 1.86						
**2. Competence satisfaction**			1.32 (.01)	9.47*** (.12)	5.36** (.07)	.59 (.01)	7.39** (.08)	5.78* (.06)
A. Competence support (CS)	2.10 ± .78	2.32 ± .69						
B. Control (C)	1.41 ± 1.27	1.74 ± 1.32						
C. Competence thwarting (CT)	1.49 ± .92	1.21 ± 1.40						
**3. Intrinsic motivation**			1.18 (.01)	9.40*** (.12)	13.07*** (.16)	1.04 (.01)	12.05** (.12)	19.44*** (.17)
A. Competence support (CS)	2.14 ± .61	2.38 ± .53						
B. Control (C)	1.64 ± 1.07	1.75 ± 1.16						
C. Competence thwarting (CT)	1.82 ± .88	1.25 ± 1.22						
**4. Performance in dribbling-shooting task (individual level)**			53.03*** (.29)	3.70* (.06)	4.66* (.07)	10.55** (.11)	2.25 (.03)	2.19 (.02)
A. Competence support (CS)	78.49 ± 9.03	73.00 ± 8.10						
B. Control (C)	78.18 ± 8.29	76.43 ± 8.76						
C. Competence thwarting (CT)	74.70 ± 7.54	71.05 ± 6.84						
**5. Performance in passing task (team level)**			35.56*** (.52)	.71 (.04)	12.13*** (.42)	8.95** (.29)	2.90 (.12)	30.52*** (.58)
A. Competence support (CS)	294.00 ± 42.80	271.17 ± 46.50						
B. Control (C)	306.17 ± 42.87	296.92 ± 47.08						
C. Competence thwarting (CT)	286.33 ± 35.58	285.50 ± 35.47						

*p < .05

**p < .01

***p < .001.

*Note*. Time 1 represents the measurement after the first test session; Time 2 represents the measurement after the second test session. The performance measures are presented in the time (seconds) needed to perform the task. The post hoc analyses represent the interaction effect of a 2 x 2 repeated measures ANOVA for each pair of experimental conditions.

It should be noted though that the perceived competence support of the leader after the first test session was already significantly higher in the competence-supportive condition in comparison with both the control condition (*t* = 3.58; *d* = .76; *p* = .001) and the competence-thwarting condition (*t* = 3.95; *d* = .83; *p* < .001). These perceived baseline differences across conditions indicate that there was less room for improvement in the competence-supportive condition, meaning that the potential positive impact of the manipulation on the outcome variables could have been suppressed.

### Primary analyses

#### Hypothesis 1: The growth-promoting and debilitating impact of the athlete leader

**Fig 2:**
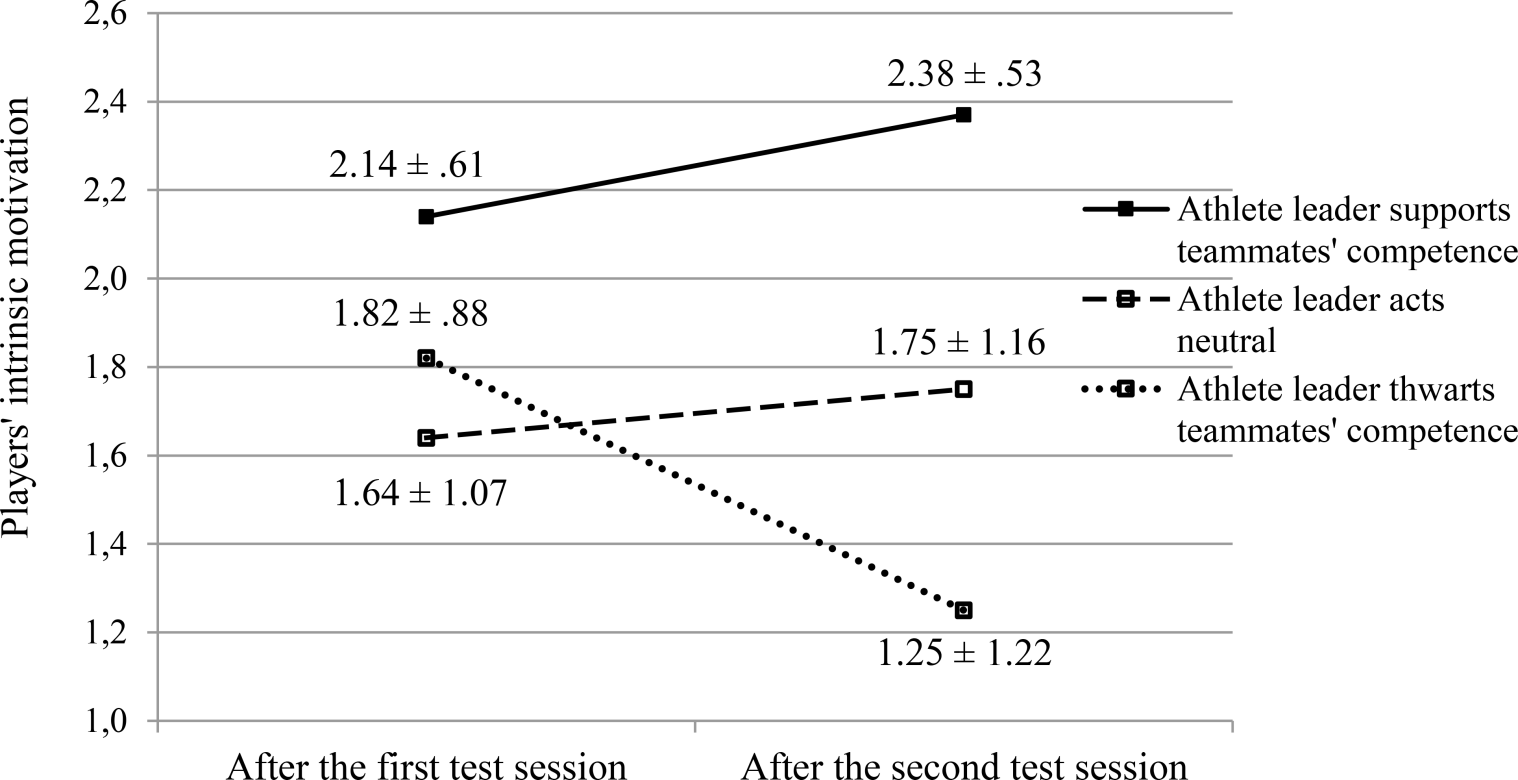
Players’ intrinsic motivation after the first and the second test sessions across the three experimental conditions.

**Fig 3:**
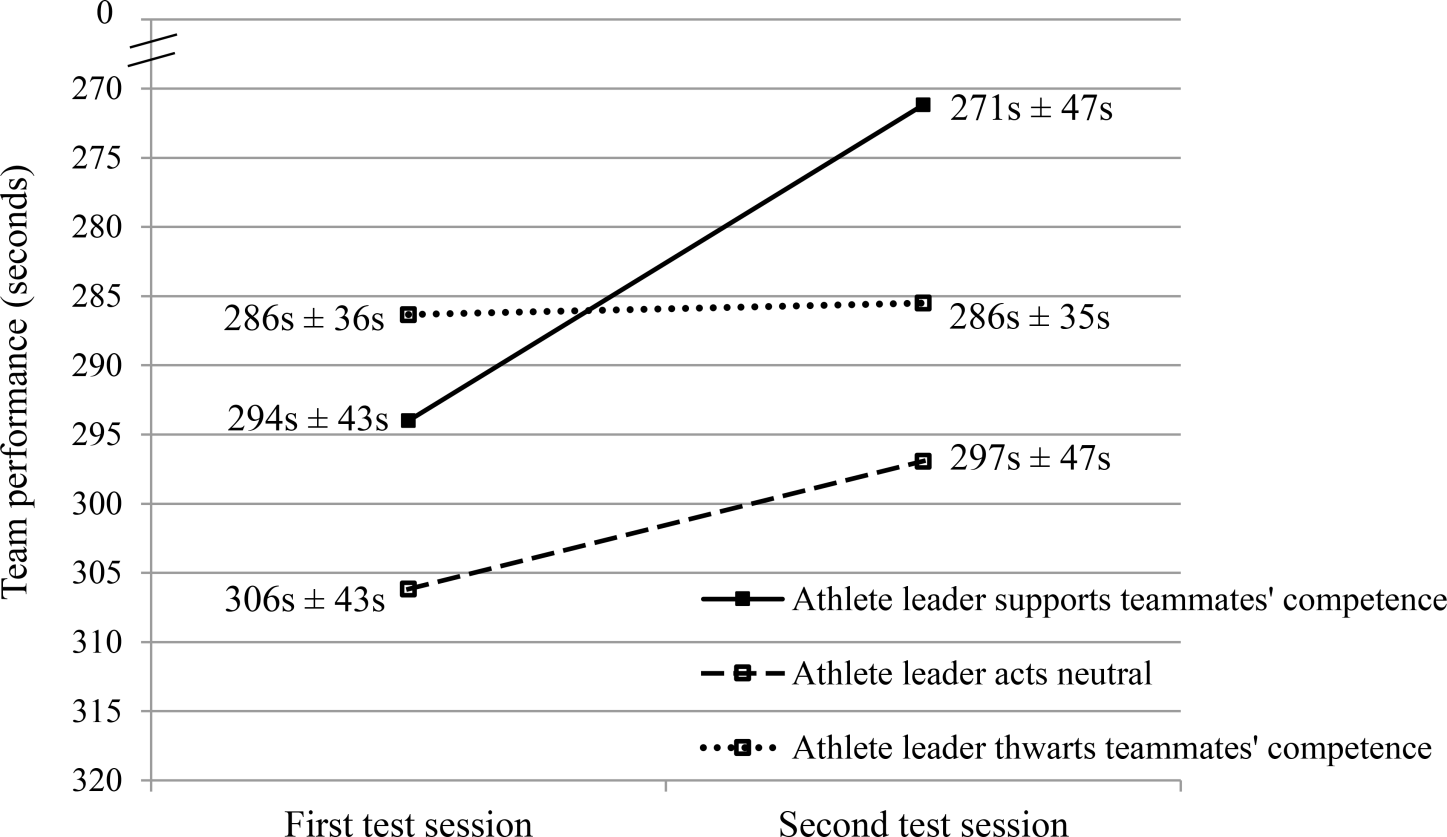
Team performance on the passing task in the first and the second test session across the three experimental conditions. The performance axis is reversed so that a higher value (i.e., less time needed to perform the task) corresponds to a better performance.

On all outcome variables, 3 x 2 repeated measures ANOVAs with the experimental condition as between-subjects factor and time as within-subjects repeated measure were performed. The results of these analyses are presented in Table 2. The findings for the two main outcome variables, namely intrinsic motivation and team performance in the passing task, are visualized in Figs 2 and 3, respectively.

#### Time and condition effects

As can be noticed in Table 2, a significant time effect emerged for both performance measures, but not for competence satisfaction and intrinsic motivation. The improvement in performance across all conditions can be attributed to a learning effect. This learning effect is particularly prominent in the control condition; although the behavior of the leader is similar in both test sessions, participants performed better in the second test session because they had already practiced the task in the first test session. This learning effect emerged both at the individual level (dribbling-shooting task) and at the team level (passing task).

The significant condition effects for competence satisfaction and intrinsic motivation can be attributed to the fact that, already after the first test session, participants in the competence-supportive condition rated these variables higher than participants in the control condition and participants in the competence-thwarting condition. This difference aligns with and is most likely caused by the difference that we found in the perceived competence support of the leader after the first test session. As a result, a ceiling effect might have suppressed the increase in competence satisfaction and intrinsic motivation in the competence-supportive condition. The significant condition effect for individual performance is smaller and can be attributed to the fact that players in the competence-thwarting condition performed better in the first test session than their peers in the competence-supportive and control condition, even though the behavior of the leader was intended to be similar in all sessions.

#### Time by condition effects

More importantly, the time by condition interaction proved to be significant for all of the assessed self-reported outcomes (i.e., competence satisfaction and intrinsic motivation). The post hoc analyses (i.e., 2 x 2 repeated measures ANOVAs) for the interaction effect found in competence satisfaction and intrinsic motivation revealed that this interaction effect arose from the difference between the competence-thwarting condition with both the control condition and the competence-supportive condition. This finding supports H2a and H2b. In other words, when the leader acted to thwart teammates’ competence, team members’ competence satisfaction and intrinsic motivation would deteriorate, in comparison to either when the leader acted neutrally or when he acted to support teammates’ competence. No interaction effect emerged between the competence-supportive condition and the control condition in the prediction of either competence satisfaction or intrinsic motivation, which partly contrasts H1a and H1b.

The pattern of findings for the performance indicators partially deviated from the one observed for the self-reported outcomes. In analogy with the effects for the self-reported outcomes, a significant time by condition effect emerged. Yet, post hoc analyses revealed that these interactions arose from the difference between the competence-supportive condition and the control condition. At the team level, the difference between the competence-supportive condition and the competence-thwarting condition also underlay the observed interaction effect. These findings are in line with H1c; when the leader behaved to support teammates’ competence, the team performance improved significantly more than when the leader acted neutrally or when he behaved to thwart teammates’ competence.

In contrast with H2c, no significant interaction effect emerged when only the control condition and the competence-thwarting condition were included in the post hoc analyses. It should be noted that, at the team level, the team performance did improve in the control condition (*t* = 2.42; *d* = .70; *p* = .03), while no significant difference emerged in the competence-thwarting condition (*t* = .26; *d* = .08; *p* = .80). In other words, the learning effect that appeared in the control condition (i.e., performance improvement without change in leader behavior) was inhibited when the leader demonstrated competence-thwarting behavior. Thwarting teammates’ competence feelings thus seems to hamper the team’s “natural” skill improvement.

#### Hypothesis 2: The explanatory role of competence satisfaction

Having detected the main effects of the manipulations, we sought to examine the potential mediating role of competence satisfaction to account for the observed effects on intrinsic motivation and performance. In order to be able to represent the three experimental conditions in our model, we took the control condition as the main reference point and created two dummy variables, with the first one representing *competence support* by the athlete leader and involving a comparison of the neutral behavior of the athlete leader (0) to the competence-supportive behavior of the athlete leader (1) and the second one representing *competence thwarting* by the athlete leader and involving a comparison of the control condition (0) to the competence-thwarting behavior of the athlete leader (1). In the following, these two contrasts are referred to as the competence-supportive and competence-thwarting contrast. Competence satisfaction and intrinsic motivation were measured after the second test session, in which the manipulation took place. The objective performance at the individual level was represented by individuals’ performance *improvement* in the dribbling-shooting task (i.e., time in the first test session minus time in the second test session). At the team level, we aggregated athletes’ competence satisfaction and intrinsic motivation scores. Furthermore, with respect to performance, we aggregated the team time on the dribbling-shooting task (excluding the individual times of the athlete leader) with the team time on the passing task. Similar to the individual level, we included the *improvement* in overall team performance in our model at the team level.

**Fig 4:**
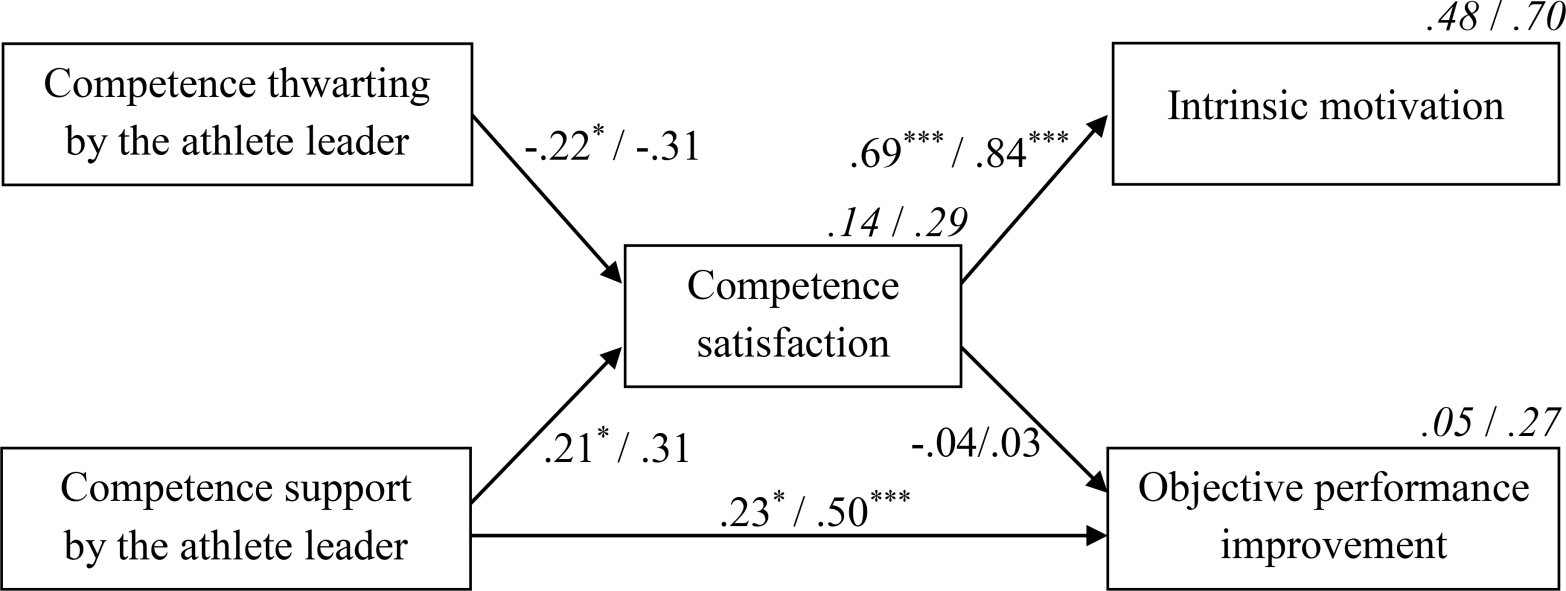
Structural model, representing the influence of competence support or thwarting by the athlete leader on intrinsic motivation and performance improvement, with competence satisfaction as mediator. Standardized regression coefficients are included (*p < .05; ***p < .001), as well as the proportions of explained variance (in italics). Both are presented as a/b where a refers to the values of the model at the individual level and b refers to the values of the model at the team level.

Given the similarity between the obtained models at the individual and at the team level, the values of both models are presented in Fig 4, with the first value of the model representing the effect at the individual level and the second value representing the effect at the team level. Goodness-of-fit indices are *χ*^*2*^ = 7.99; *p* = .09; *CFI* = .96; *TLI* = .92; *RMSEA* = .09; *pclose* = .19 at the individual level and *χ*^*2*^ = 6.39; *p* = .17; *CFI* = .96; *TLI* = .91; *RMSEA* = .14; *pclose* = .21 at the team level.

As can be noticed in Fig 4, both experimental contrasts were uniquely related to competence, with the competence-supportive contrast yielding a positive impact (β = .21 at the individual level and β = .31 at the team level) and the competence-thwarting contrast yielding a negative impact (β = -.22 at the individual level and β = -.31 at the team level). Furthermore, in line with our hypothesis, competence satisfaction significantly predicted the intrinsic motivation of both individual athletes (β = .69) and the team as a whole (β = .84).

In addition to the direct effects reported in Fig 4, Table 3 represents the indirect and total effects at the individual and at the team level. An examination of the indirect effects on intrinsic motivation revealed that, in line with H3a, competence satisfaction mediated the relationship between leader’s competence supportive behavior and teammates’ intrinsic motivation. This hypothesis was further confirmed given that the competence-thwarting behavior of the athlete leader negatively affected intrinsic motivation via reduced feelings of competence. While these indirect effects were significant at the individual level, they were only marginally significant at the team level (*p* = .08). Considering that the effect sizes at the team level were moderate and even exceeded the effect sizes at the individual level, this non-significance should probably be attributed to the limited number of teams compared with the number of individual athletes.

**Table 3:** Indirect effects (IE), total effects (TE), and standard errors (SE) for all paths in the postulated model both at the individual and at the team level between predictors (in rows) and outcomes (in columns).

		*Competence satisfaction*		*Intrinsic motivation*		*Performance improvement*	
		*Effect*	*SE*	*Effect*	*SE*	*Effect*	*SE*
**Model at the individual level**							
Competence thwarting by the athlete leader	IE			-.14*	.06	.01	.02
	TE	-.22*	.10	-.14*	.06	.01	.02
Competence support by the athlete leader	IE			.13*	.06	-.01	.02
	TE	.21*	.10	.13*	.06	.21*	.08
Competence satisfaction	TE			.64***	.06	-.04	.09
**Model at the team level**							
Competence thwarting by the athlete leader	IE			-.24	.14	-.01	.05
	TE	-.31	.18	-.24	.14	-.01	.05
Competence support by the athlete leader	IE			.24	.13	.01	.05
	TE	.31	.17	.24	.14	.50**	.15
Competence satisfaction	TE			.77***	.09	.03	.17

Note.

*p < .05

**p < .01

***p < .001.

With respect to the performance pathway, we also hypothesized that competence satisfaction would act as an underpinning mediator explaining the impact of athlete leaders. However, this was not the case. Whereas the competence-supportive and competence-thwarting behavior of the athlete leader significantly predicted athletes’ competence satisfaction, competence satisfaction did not predict performance (β < .05). Instead, a direct effect from the leader’s competence support on performance had to be added to improve the model fit. Doing so resulted in a significant change in model fit (*p* = .02 at the individual level and *p* = .005 at the team level, respectively). By supporting their athletes’ competence feelings, coaches can thus directly impact their athletes’ individual performance (β = .23), as well as the team’s overall performance (β = .50). This direct effect did not emerge for the leader’s competence-thwarting behavior.

## Discussion

The present study provides experimental support for the impact of the athlete leader on teammates’ intrinsic motivation and performance by supporting or thwarting their need for competence. The study findings highlight the importance of leaders *within* the team for optimal team functioning, thereby corroborating previous research on the crucial role of athlete leaders (for a review, see [27]).

### The growth-promoting and debilitating impact of the athlete leader

Consistent with our hypotheses, the findings of this study revealed that when the athlete leader acted in a competence-supportive way by providing positive feedback and expressing confidence, his teammates became more intrinsically motivated to engage in the exercises than when the leader acted to thwart teammates’ feelings of competence. Furthermore, the study findings revealed that athlete leaders did not only impact teammates’ intrinsic motivation, but also their performance. Indeed, when the athlete leader behaved to support his teammates’ feeling of competence, the team made significantly greater performance improvements compared with when the leader acted neutrally or when he acted to thwart teammates’ competence feelings. These findings were consistent at both the individual and the team level, thereby providing experimental support for previous cross-sectional results that highlighted the various beneficial outcomes of a competence-supportive environment, including motivation and performance [12, 15]. Because intrinsically motivated athletes show more enjoyment, higher satisfaction, maintained effort, adherence to their sport, and a greater persistence [3], athlete leaders have the ability to significantly improve the team’s functioning.

However, athlete leaders can also negatively impact their team. Indeed, the present study extended previous literature by investigating not only the growth-promoting potential of a competence-supportive leader, but also the motivational pitfalls and downsides associated with a competence-thwarting leader. In doing so, our findings provided experimental evidence on the negative impact of athlete leaders on their teammates’ motivation and performance when thwarting their competence feelings. Although the observed effects mainly confirmed our hypotheses, it should be noted that not all the individual effects were in line with our expectations.

First, with regard to both competence satisfaction and intrinsic motivation, no interaction effect was found between the competence-supportive condition and the control condition, suggesting that the provision of athlete competence support did not boost these outcomes relative to a control group (although it did relative to a competence-thwarting approach). A possible explanation is that due to a learning effect, participants’ performance improved during the experiment, also in the control condition when the leader’s behavior remained the same. This performance improvement has most likely increased participants’ feelings of competence satisfaction, their enjoyment, and hence their intrinsic motivation in the control condition. However, one would expect that the competence-supportive condition would show an even greater increase in intrinsic motivation, which was not the case. The underlying reason may be that participants’ initial intrinsic motivation and competence satisfaction were already elevated in the competence-supportive condition compared to the control condition, probably because of the initial perception of a more competence-supportive leader at the start of the experiment in the competence-supportive condition. Because participants’ initial intrinsic motivation was already close to the maximum of the scale (i.e., 2.14 on a scale of -3 to 3), a ceiling effect might have left little room for further improvement during the experiment in the competence-supportive condition. As a result, a similar increase as in the control condition emerged, and hence no interaction effect could appear. Such an interaction effect did appear between the control condition and the competence-thwarting condition, in which participants’ competence satisfaction and intrinsic motivation deteriorated along the experiment.

Second, with regard to performance, it should be noted that, although a significant interaction effect emerged between the competence-supportive and control condition both at the individual and at the team level, no interaction effect was found between the competence-thwarting and control condition. A more detailed analysis revealed that in the dribbling-shooting task, the performance improvement in the competence-thwarting condition was not significantly different from the learning effect in the control condition. By contrast, in the passing task, the learning effect that was apparent in the control condition was inhibited when the leader thwarted teammates’ competence.

This contrasting effect can possibly be explained by *social loafing*. Social loafing refers to a reduction in motivation and effort when people work in a group compared with when they work individually [45]. Research on social loafing has pointed out that the visibility of individual performances in a team task is a crucial predictor of social loafing [46]. More specifically, when players feel as though their level of effort cannot be ascertained because the task is a collective one, social loafing becomes more likely. In contrast, when people feel that their individual performance can be evaluated, they tend to exert more effort and their productivity increases [47].

The different nature of the two tasks in our experiment possibly may have caused the contrasting effect of competence thwarting on performance. More specifically, the dribbling-shooting task is a low-interaction task, in which the individual performances can be isolated and are aggregated into one team score. Although the competence-thwarting behavior by the athlete leader resulted in motivation losses (i.e., significant decrease in intrinsic motivation), no difference was observed in performance improvement compared with the control condition. The fact that the individual performances could be identified in the dribble-shooting task might have inhibited social loafing on this task. By contrast, the passing task involves high interaction between the players, as a result of which the individual performances could not be isolated. Consequently, social loafing is more likely to occur on this passing task. Our findings revealed indeed that the competence-thwarting behavior by the leader negatively affected the team performance in the passing task.

### The explanatory role of competence satisfaction

In line with our hypotheses, structural equation modeling showed that the relationship between both competence support and competence thwarting by the athlete leader and intrinsic motivation was accounted for (i.e., mediated) by competence satisfaction. The present findings corroborate previous research in the educational environment, which revealed that positive feedback satisfied students’ need for competence, thereby enhancing students’ intrinsic motivation [8, 11, 48]. Furthermore, our findings experimentally confirmed earlier cross-sectional and longitudinal findings in a sports setting showing that perceptions of competence predict players’ intrinsic motivation [13, 14]. With regard to competence thwarting, the present study extended the cross-sectional findings of Bartholomew, Ntoumanis, et al. [17] by experimentally demonstrating that need thwarting by the leader was negatively related to competence satisfaction.

In contrast to our hypotheses, the relation between both competence support and competence thwarting and performance was not mediated by competence satisfaction. A number of explanations can be provided. First, in the current study we only assessed team members’ competence satisfaction and not their competence frustration. Vansteenkiste and Ryan [16] emphasized that competence frustration is more than a lack of competence satisfaction. Indeed, competence frustration becomes apparent when athletes’ need for competence is actively thwarted and may have severe adverse effects. It is therefore possible that competence frustration would have acted as a mediator in explaining the athlete leader’s impact on team members’ performance.

Second, the competence-supportive condition yielded a direct performance-boosting effect, which was not driven by shifts in competence satisfaction. The athlete leader’s expression of confidence and the delivery of motivational feedback may have prompted teammates to put extra effort in the activity, thereby executing the task more quickly. Also, given that competence was assessed after task completion (rather than midway), it is well possible that performance improvement may have caused shifts in competence satisfaction, thus pointing towards a bidirectional association. Indeed, athletes may have inferred that they are more competent in executing the activity based upon their faster task execution. By the inclusion of multiple assessments of competence and performance, future research may want to shed light on the bidirectional relation between both.

Third, a final explanation is that other mechanisms than competence satisfaction or frustration come into play. The Social Identity Approach (SIA; [49]) provides an additional framework to explain the direct link between competence support and performance. This theoretical approach asserts that people’s sense of self can be defined in terms of their personal identity (i.e., in terms of ‘I’, as unique individuals), but also in terms of their social identity (i.e., in terms of ‘us’, as group members who share goals, values, and interests with others). In its recent application to leadership [35], SIA argues that leaders are able to exert influence on their team members to the extent that they manage a collective sense of ‘us’. Two previous experimental studies, within a basketball and soccer context respectively, revealed that the identification with the team was the mechanism underpinning the team confidence contagion, emanating from the leader [31, 34]. In other words, when the leader expressed confidence in his team (which is a competence-supportive behavior), teammates more strongly identified with their team, became in turn more confident in their team’s abilities, and ultimately performed better. In line with these findings, we suggest that the creation of a shared sense of ‘us’ is an alternative mechanism to explain the impact of the leader’s competence-supportive and competence-thwarting behavior on team members’ performance.

### Strengths of the present study

The present study is the first to demonstrate the effects of competence-supportive and competence-thwarting behavior by the athlete leader on the intrinsic motivation and performance of his teammates. The study findings thus show that earlier findings in organizational, educational, and sports setting (i.e., with regard to competence support by the coach) also apply for athlete leaders, thereby further extending the application area of the Self-Determination Approach [8].

Another strength of the present study constitutes the experimental study design, which also included a control condition in addition to the competence-supportive and competence-thwarting condition. As such, the design improved previous experimental designs (e.g., [31]), thereby providing a deeper insight with respect to which effects can be ascribed to a learning effect.

Furthermore, in contrast with previous laboratory experiments, using standard motor tasks (e.g., [22, 50]), the present study attempted to obtain a high external validity by performing the experiment on the soccer field of the participants. More specifically, we used two soccer drills that consisted of specific motor tasks that characterize a soccer game (i.e., dribbling, shooting, passing). To further improve the resemblance with a real soccer game, we also included a passing task that required high interaction between the players, in contrast to the more indivualized tasks that were used before (e.g., the free throw shooting task in the study of Fransen, Haslam, et al. [31]).

### Limitations and avenues for further research

Because we strived to optimize the internal validity of this standardized experimental study, some limitations with regard to the external validity were inevitable. For example, to rule out prior familiarity between participants, we created new teams in which the players were not familiar with each other in advance. Furthermore, the athlete leader in the team (i.e., a research confederate) was unknown to the other players. It can be expected that in a real soccer team, in which players know each other well and the athlete leader has earned his leader status through long-term interactions with his teammates, the impact of the athlete leader is even more powerful. This argument is well illustrated by a follow-up experimental study that examined the impact of the actual athlete leader in basketball teams (i.e., the athlete that was rated by his teammates as being the best leader) instead of using a research confederate [51]. The study findings demonstrated that the provision of motivational feedback by the athlete leader increased teammates’ competence satisfaction, intrinsic motivation, and objective performance, relative to a control group. Noteworthy is that the observed significant interaction effects (competence support vs. control) for competence satisfaction and intrinsic motivation were not found in the present study. This difference suggests that the present study findings, using a research confederate acting as an athlete leader, underestimate the impact of the actual athlete leader in the team. Furthermore, the results of the follow-up study confirmed our current results as competence satisfaction mediated the motivational pathway (i.e., leaders’ impact on athletes’ intrinsic motivation), while a direct impact was observed for the performance pathway.

A second limitation is that the present study only focused on the impact of athlete leaders on their teammates, and not on the influence the other players can exert on each other. The Social Learning Theory [52] posits that people learn from one another, via observation, imitation, and modeling. In this regard, athlete leaders may act as role models for their teammates, who might in turn imitate their leader’s behavior [53, 54]. Consequently, the competence-supportive or competence-thwarting behavior by the athlete leader might instigate a team atmosphere in which all the players start supporting or thwarting each other’s competence. It goes without saying that such an atmosphere can lead to much stronger effects than those obtained in our study. A longitudinal study would provide more insight in the contagion of competence-supportive and competence-thwarting behaviors, as well as in the associated reciprocal effects.

### Practical implications

The study findings highlight the importance of athlete leaders in fostering teammates’ intrinsic motivation and performance. However, the present findings also emphasize the risk of athlete leaders undermining teammates’ intrinsic motivation by thwarting their need for competence. Therefore, coaches would benefit from guiding their athlete leaders well and teaching them how to support the competence of their teammates in order to improve the team’s functioning.

A first step in this leader development process would be to identify the athlete leaders in the team. In this regard, it should be noted that the athlete leader is not always the captain of the team. Instead, often the informal leaders (i.e., athlete leaders without formal leadership recognition who receive their leader status through natural interactions with their team members) are often perceived as the real athlete leaders of the team [6]. Social network analysis has very recently been proposed as a valuable tool to obtain a deeper insight in the leadership structure of sports teams [7]. Based on the perceptions of all the players in the team, social network analysis identifies the players in the team that are perceived by their teammates as “best” athlete leaders. The fact that a leadership role is not imposed by the coach but rather chosen and accepted by all the players within the team can increase athlete leaders’ commitment to their role as well as the effectiveness of their interventions.

After identifying the athlete leaders in the team, coaches do well to inform leaders on their function as role model for their team. To fulfill this role model function well, coaches have to teach their leaders how to act competence-supportive in all circumstances and how to avoid any competence-thwarting behavior. In this way, coaches can use the power of their athlete leaders to establish an optimal team environment, in which players’ competence satisfaction and intrinsic motivation is bolstered and their progress is accelerated.

## Supporting information

S1 Table Dataset including variables at the individual level. (SAV)

S2 Table Dataset including variables at the team level. (SAV)
